# How are health professionals earning their living in Malawi?

**DOI:** 10.1186/1472-6963-6-97

**Published:** 2006-08-09

**Authors:** Adamson S Muula, Fresier C Maseko

**Affiliations:** 1 Department of Community Health, University of Malawi, College of Medicine, Blantyre, Malawi; 2 Department of Basic Studies, Malawi College of Health Sciences, Lilongwe, Malawi

## Abstract

**Background:**

The migration of health professionals from southern Africa to developed nations is negatively affecting the delivery of health care services in the source countries. Oftentimes however, it is the reasons for the out-migration that have been described in the literature. The work and domestic situations of those health professionals continuing to serve in their posts have not been adequately studied.

**Methods:**

The present study utilized a qualitative data collection and analysis method. This was achieved through focus group discussions and in-depth interviews with health professionals and administrators to determine the challenges they face and the coping systems they resort to and the perceptions towards those coping methods.

**Results:**

Health professionals identified the following as some of the challenges there faced: inequitable and poor remuneration, overwhelming responsibilities with limited resources, lack of a stimulating work environment, inadequate supervision, poor access to continued professionals training, limited career progression, lack of transparent recruitment and discriminatory remuneration. When asked what kept them still working in Malawi when the pressures to emigrate were there, the following were some of the ways the health professionals mentioned as useful for earning extra income to support their families: working in rural areas where life was perceived to be cheaper, working closer to home village so as to run farms, stealing drugs from health facilities, having more than one job, running small to medium scale businesses. Health professionals would also minimize expenditure by missing meals and walking to work.

**Conclusion:**

Many health professionals in Malawi experience overly challenging environments. In order to survive some are involved in ethically and legally questionable activities such as receiving "gifts" from patients and pilfering drugs. The efforts by the Malawi government and the international community to retain health workers in Malawi are recognized. There is however need to evaluate of these human resources-retaining measures are having the desired effects.

## Background

The shortage of health professionals in almost all of the African health systems has received international recognition [1-3]. African health systems already inadequate to deliver health services to meet the goals of Primary Health Care (PHC) as agreed in Alma Alta in 1978 and the Millennium Development Goals (MDGs) have been overstretched even more with the demands and consequences of the HIV and AIDS pandemics [4,5]. Although outward migration of Africa's professionals is not a new phenomenon, and has been occurring due to several years, the effects of the migration in the past several years has been unprecedented. The reasons behind the migration of health professionals from Africa to mostly developed nations, especially Australia, Canada, the United States, United Kingdom and New Zealand, have been described [6-8]. These have been categorized as "push factors" and "pull factors". The "push factors" are those factors operant in the African "donor" country from which health professionals are living. The "pull factors" are the green pastures and other activities that influence recruitment in the "recipient" western developed nations [3]. The Regional Network for Equity in Health in East and Southern Africa (EQUNET) have described the migration of African health professionals to northern countries as "reverse subsidy", as African countries are increasingly becoming a formidable and unwilling donor community of health professionals to developed nations, many of whom are members of the G7 [9,10].

Although the issue of Africa health professionals' migration to northern countries has received attention by the research and academic community, policy makers and the wider community, oftentimes the discussion weighs more on what are the issues that are responsible for this "brain drain" of Africa's resources. Not that this is not important, but rather it is just part of the whole picture. In most African health systems, the proportion of health professionals that have migrated out of the continent, although a large number, are still the minority of all the health professionals that the concerned countries would have had. Not much attention has been spent on describing how those health professionals that have not left their posts in Africa are earning their living. In order to contribute to the literature on the African brain drain, we conducted a qualitative study of Malawi as a case study, with the following specific objectives: a) determine the sources of income for health professionals in both private and public services in Malawi other than formal wages (pay salaries); b) determine working practices of health professionals in Malawi that may influence their retention in the country; c) determine attitudes of health professionals towards various forms of out of formal employment incomes; d)identify possible strategies that could contribute to retention of staff and have a positive impact on the problem of brain drain in Malawi.

## Methods

This study utilized focus group discussions and in-depth key informant interviews. The study participants included health professionals and administrators working at health centres, district level as well as the Ministry of Health (MoH) headquarters. In total, 35 nurses, 15 doctors, 25 clinical officers, 15 medical assistants, 40 technical support staff and 15 administrators and policy makers were interviewed. This method was deemed necessary for collecting data in an exploratory way as has been described by Morse [11].

Study participants were identified both purposively and through convenience sampling. At the level of the Ministry of Health and hospital management study participants were mainly identified purposively as participants were interviewed by virtue of their position. Other health professionals were interviewed based on their availability at the time of visit to the health facility. At the health facilities visited the officer-in-charge, matrons and ward sister-in-charge were requested to assist in identification of potential participants. This would have introduced bias as the persons assisting in identification of potential participants may have favoured or selected against certain individuals. However, noting that even if that were the case, themes obtained were repeated at distant sites may suggest that the information obtain could be generalized to No attempts to interview were made towards health professional who were not available at time of interview. Health professionals from all three regions of Malawi (south, center and north) were interviewed. Interviews were noted on paper and were not recorded the perceived sensitivity of the topic needed assurances that participants identities could not be inadvertently revealed. Data were analysed manually based on literature reviewed and emerging from the interviews based mainly on challenges being encountered, efforts to cope within such an environment and the perception held towards those survival mechanism. This was done by identifying repetitive. Literature was searched at the study design stage to guide in formulation of relevant questions for the interview guides.

### Ethics and participants consent

Overall permission to conduct the study was obtained from the Ministry of Health and Population (MoHP). At each of the sites visited, the officer-in-charge also gave assent after being explained the objectives of the study. Study participants gave verbal informed consent. Participants' details were recorded only by their designation e.g. nurse, clinical officer and no names were recorded.

## Results

Various themes emerged from the interviews and the literature searches on the retention and survival mechanisms of health professionals in Malawi. In general, the following themes were identified; challenges faced health professionals, survival strategies and perceptions towards the survival strategies. The sub-themes under challenges faced included; inequitable salaries and recognition of experience, overwhelming responsibilities, lack of stimulating interaction in the workplace, inadequate supervision, impact of HIV and AIDS, limited access to further training, limited career recognition, lack of transparency in recruitment of staff, and discriminatory remuneration. The sub-themes under survival strategies included ways of minimizing expenditure and increasing income.

survival strategies which included efforts to increase income and means to reduce personal and household expenditure.

### Challenges faced by health professionals

Challenges faced by health professionals in Malawi fall into those that impact on work status and remuneration, working conditions, opportunities for further training/advancement and unfair recruiting practices.

### Inequitable salaries and recognition of experience

In case of medical doctors, when the College of Medicine's first graduates completed their internships in 1994 all of them were sent to districts to work as district health officers (DHOs). This was the first largest group of Malawian doctors to be deployed to districts at any one time. This practice continues and newly qualified doctors upon completing their internships are posted as DHOs. This however creates a situation where much more experienced DHOs who have worked for several years may be at the same grade as those just coming in with no experience, and remunerated at the same grade with the same privileges. A doctor who had stayed in a rural district post for many years said:

"I have served as a DHO for many years. Someone has just come in from internship. We are at the same grade. What is that?"

This was perceived by the long-serving doctor as unfair and de-motivating. There was perception that doctors coming in straight from medical school ought to be at a lower scale than those that had served in their posts for several years.

Nurses felt that they were marginalized by the MoH and other employers in favour of medical doctors, clinical officers and medical assistants. Of particular note, in Malawi is that these other health cadres (clinical officer, medical assistant) are predominantly male dominated while the nursing field is female-dominated [12,13]. For instance, doctors within the Christian Health Association (CHAM) health facilities were eligible for salary supplementation from the Germany government's Deutsche Gessellschaft fur Technische Zusammenarbeit(GTZ) Gmb funding which may be in excess of US$ 300 each month, nurses on the other hand were not accorded no such privileges.

While DHOs were in many cases doctors and in a few cases clinical officers, it seemed that no nurse, even those with a degree, could be appointed a DHO. Interestingly though, clinical officers, in some cases not as well trained as the degree nurses, could be DHOs. This inevitably resulted in discontent among degree nurses who ended up being subordinates to clinical officers and medical assistants. Registered nurses also felt humiliated in situations where they were subordinates to medical assistants, who had two years of professional training as opposed to the degree nurses' four to five years. A newly graduated medical assistant (MA) could be sent to a health centre and be in charge of that health center, responsible for all the supplies and operations at that unit, including supervising the nurse, (in some cases a registered nurse) who may have been at the health facility for many years.

As one nurse said:

"*What is painful is that even myself as a degree nurse must work under a medical assistant who has a mere two year certificate."*

Situations like those stated above were reported to be sources of frustration and resentment towards the health care system.

### Overwhelming responsibilities

Health professionals in Malawi reported finding themselves taking up responsibilities that were beyond what their training and/or experience had equipped them for. In many other cases, the equipment or supplies required to enable them perform their duties so as to deliver high quality health services were not available. In the case of doctors who complete their internships and desire to continue working in the public health sector, many are sent to work as DHOs expected to provide both clinical and administrative leadership for an entire district, with little experience for such challenging situations. Some districts have population greater than 400,000 but still with fewer health workers and facilities to enable full coverage of services.

The situation was little different for degree nurses who are sent to district hospitals. There are just a few nurses that are sent to rural districts and many actually serve at the district hospital itself. The degree nurse is likely to be appointed a ward sister-in-charge, an acting matron or in some cases even a matron, working as the district nursing officer. While the health professional is trained at KCN to take up such responsibilities at the appropriate time after having acquired the requisite experience, the urgency to take on such responsibilities without first having gained experience can easily lead to frustration and burn out.

While study participants recognized that being put in administratively demanding jobs was a problem, they also recognized that it was difficult not to appoint degrees nurses or the only medical doctor in the district to very senior positions. Related to this was the fact that invitations to workshops, seminars and conferences, and therefore opportunities to access allowances/per diems usually went to these senior cadres. The new degree nurse may therefore feel pressured to attend to all these meetings, thus leading to burn out.

### Lack of stimulating interaction in the workplace

Health workers in the public sector in rural Malawi reported working in isolation due to the low number of other health professionals in the same locality. It was not uncommon to have the only doctor in a particular district as the DHO, and many districts having the only laboratory technician or radiographer with no other employees in the same profession. There were only a few registered nurses in each district, and the situation was similar for other cadres. In many cases the professional isolation implies an environment that was not challenging due to a lack of peer support and sharing of ideas, resulting in frustration because of the lack of professional interaction. The lack of academically and professionally challenging environment was exacerbated by the absence of any requirement for continued professional development (CPD) or professional medical education (CME). CME is a process operation in many countries, especially developed nations where the health professional is required by law to demonstrate active acquisition of knowledge or updating one's skill in order for re-registration by the regulatory and professional bodies to occur. As there was requirement for continued professional development as a necessity for re-registration by the regulatory bodies, health workers felt there were not being "pushed" to maintain excellence.

### Inadequate supervision

Supervision is perceived to be a motivating factor by health workers. Workers in this study reported they were either poorly or inadequately supervised. Those that were supervisors reported that lack of resources and especially transport to rural remote areas coupled with other commitments and responsibilities prevented them from making supervisory visits. In some cases lack of the requisite training and experience in undertaking supervision seemed to result in poor understanding and appreciation of the importance of supervision. It was felt that in order not to expose their shortcomings, some supervisors at times desisted altogether from undertaking any supervision. Lack of supervision, resulting in workers feeling unappreciated, de-motivated and frustrated, was an acknowledged reason health workers were not interested to work anymore within the public health sector.

### Impact of HIV and AIDS

There was a general perception among health professionals that they were at an increased risk of occupational exposure to HIV and other infectious diseases, such as tuberculosis. Although almost all health facilities had a focal person responsible for universal precautions against hospital acquired infections the availability of supplies such as disinfectants was not universally guaranteed.

Apart from occupational exposure to HIV, it was likely that a significant number of health workers were infected with HIV although HIV prevalence figures among health workers in Malawi are not available. In 1999, the Health Sector Human Resources Plan reported the following losses due to death: registered nurses (2.7%), clinical officers (2.1%), medical assistants (2.1%), and 1.9% for all enrolled nurses/midwives [14]. Although the Health Sector Human Resources Plan did not indicate the distribution of causes of deaths among the various health cadres, AIDS is likely to be an important factor. Shisana et al reported that HIV prevalence among health workers in South Africa was 15.7%, with younger health workers (18–35 years) having a much higher prevalence of 20% [15]. Tuberculosis infection rates among health workers in Malawi are higher than the general community [16]. While there is all likelihood that tuberculosis acquisition in caring of patients is higher for health workers, many people do not normally think that the suspected high rates of HIV among health workers is due to transmission from patients to health workers.

The heavy work load coupled with the perception of increased risk of getting an infection (HIV, tuberculosis), result in some health professional changing from clinical to other duties, or leaving the health profession altogether.

### Limited access to further training

Many health professionals valued access to further training. Employment as a junior faculty member within a university department (at the Kamuzu College of Nursing and the College of Medicine) almost always guarantees the opportunity to obtain a postgraduate qualification, either from the institution itself or from another institution. Sponsoring agencies and government are more likely to support postgraduate training for a university employee than for a health professional working in a non-educational institution. This offered as an attraction for employment in the training institutions for those health professionals who desired to obtain higher qualifications.

Tutors in health training colleges are normally employed when they have three year diploma and they thus have an opportunity to obtain advanced or higher diplomas as well as degrees. In the case of the Malamulo and the Malawi College of Health Sciences, one of the challenges however is the fact that many of these degrees obtained by tutors are not in mainstream clinical training but rather in areas like health education. Such qualifications are mostly good for the teaching position but little recognized by the public sector to facilitate advancement in mainstream clinical jobs.

Previously with limited numbers of health professionals competing for training posts, the pubic health sector was competitive in as far as providing or facilitating training fellowships/scholarships. While this continues to occur, there are many more health professionals requiring such training and demand can hardly be met.

Unfortunately it would seem also that blocks are occurring at MoH headquarters and that some do not support the provision of postgraduate qualification to MoH employees. A case in point would be the handling of World Health Organisation's training fellowship from the WHO's Malawi office. Malawi should receive, up to 20 WHO-funded training fellowships every two years from the WHO Malawi office. Despite the availability of this facility, the MoH has dragged its feet since 2002, impeding Malawian health workers' ability to benefit from such opportunities, and thereby removing a reason to remain within the public health sector.

The College of Medicine has, from 2005 started providing specialist degree course in several the core clinical specialties of Internal Medicine, Surgery, Obstetrics and Gynaecology and Surgery. Graduate public health training was started much earlier in 2003 [17]. Currently however this training in the clinical specialist programs is only available to employees of the MoH and College of Medicine. Private sector employees are not eligible. This may be an incentive for doctors to work in the public sector. However, for those in the private sector, the only opportunity for training is available outside the country.

### Limited career recognition

Access to further training can be a motivating factor assisting in the retention of health professionals. However, when a health professional has acquired additional qualifications, they require appropriate recognition for their achievement. In several instances, the MoH does not seem to accord this expected recognition to a staff member. Frustration and resentment set in and the health professional may leave. Several medical doctors obtained post-graduate qualifications abroad in Public Health/Epidemiology. Upon return, these health professionals, who had left as DHOs, seemed to have expected promotion to higher MoH positions. When this did not happen, a few stayed on leave for several months while they negotiated with the MoH for their posting while others resigned.

The MoH is not solely responsible for the lack of recognition for its staff since it can only promote its staff to P5 grade, a level already attained by several DHOs. Promotions above this grade are the prerogative of the Office of the (State) President, i.e. political appointments. In essence, what the system can do is that it is possible for a health professional to be promoted to P5 grade and beyond that it is the State President's who must do that.

Coupled with the need for further promotions to be effected by the Office of the President, established posts may not be available for an employee who has upgraded themselves. Currently, the posts within the civil service are established centrally, with significant guidance from the Department of Human Resources (DoHR) of the central government. The DoHR receives many requests from all government departments for creation of new posts. It must consider both the short-term and long-term financial implications of creating new posts. In many cases, establishment of new posts can not be created and a health worker who has acquired further training may have to be wait until the holder of the position leaves the civil service in any way.

### Lack of transparency in recruitment of staff

There was a perceived lack of transparency in the recruitment of health professionals. Health professionals reported that even in some public institutions there were no public advertisements for posts but rather a process of headhunting had occurred. A doctor reported:

"What you first hear is that there is a vacancy and so and so has been earmarked for such a post. At first, you think it is not true. Let me apply for the post. You are not successful but to your surprise, the candidate whom people said would take the post is the successful one. Now I don't think I can apply for a job there anymore."

Even when the advertisements are placed there is still an expectation that an individual has been earmarked for the post. This suspicion was reported as often proven correct with the identified individual being recruited. Among the reasons given for the absence of public advertisements by the employing institutions was the lack of money for adverts and the reasoning that "*even if you advertise, people will still not apply for the post."*

Whatever the reasons behind such a practice, some health professionals indicated that such practices are de-motivating. Many just remain in the public service in order to gain work experience before moving on [18].

### Discriminatory remuneration

Respondents indicated that one area that they find frustrating is in receiving much lower remuneration packages than expatriate technical support staff who in some cases may have the same or even a lesser qualification and experience. Respondents noted that they could understand "reasonable" differences in remuneration between national and expatriate staff *"up to a degree but not to a level where one is tempted to think that nationality matters"*.

### Survival strategies for health workers

Health workers have adopted various strategies ranging from what can be described as legal through the unethical to criminal behaviours to survive poor conditions and low pay. A number of strategies require the health worker to take on additional responsibilities either in or outside the health sector, while others require varying degrees of personal denial. Several of these coping strategies do occur as a result of inefficiencies within the health system while some are only possible because of poor governance. The survival strategies can broadly be grouped into two categories i.e. increasing income and minimizing expenditure.

### Increasing income

In this category, health workers' actions that provide them opportunity to earn more will be discussed.

### Working closer to home villages

Many Malawians working in the formal employment sector are also involved in farming. Some health professionals, even when they do not work in their home districts choose to work in a district close to their home village. This accords them opportunity to farm in their home districts should they wish to do so. Farming was identified as one way for health professionals to earn extra money through the sale of farm produce; to reduce expenditure on food; and to support relatives who may be provide labour on the farms.

### Stealing drugs

It is often reported that significant quantities of drugs supplied to the public health sector end up being pilfered and sold. While unknown robbers are among the culprits, some health workers are reported to be involved in stealing drugs and selling them to drug vendors or private clinics [19]. Some pharmacy assistants and technicians in particular are reported to be heavily involved. This practice has been reported in many other African countries [20].

In order to curb the diversion of drugs the government introduced labeling of drug tablets with the letters "MG". However, this has not had the required effect, and drug thefts continue to be discharged at the courts "for lack of sufficient evidence" even when culprits have been caught with tablets with "MG" labels. An administrator in the MoH said:

"Much of the drugs are stolen by pharmacy personnel themselves. This is a fact. Now, even when you catch someone red-handed, it does not get very far. The culprit gets bail the next day and you can never win a case like that. Even the judges and the lawyers say the MG on the tablet could mean Margaret Gama. This is absurd."

Another administrator was rather sympathetic:*"It ought to be known that if you cheat when paying someone, s/he will also cheat you when s/he can. The pharmacy staff are stealing medicines, the administrators are running from one workshop to the other while some don't report for duty. Pay them well and these evils will reduce."*

The practice of stealing drugs as a known occurrence within the Malawi Ministry of Health has also been reported by Chaulagai et al [21]. These authors reported that at workshop by the MoH, the Minster mentioned about the issue but did not address the matter significantly although the MoH was aware of which health professionals were culprits in this practice.

### Short-term and long training

Health professionals who enroll in either short-term or long-term sponsored training programs have the opportunity to enhance their financial situation. These training programs come with reasonable living allowances that enable the employee and their family to live comfortably and may contribute to the acquisition of household property and/or fixed assets.

A lack of promotional opportunities for enrolled nurses has resulted in some training as psychiatric nurses or as anesthetic clinical officers. An enrolled nurse who trains as a psychiatric nurse is promoted from her TA grade to a TO (diploma grade). However many trained psychiatric nurses are no longer providing specialist psychiatric care but rather are employed in units or hospital departments providing routine care where there may not be as much need for specialist psychiatric training. In contrast, many medical assistants who have benefited from training in orthopaedics and anesthesia contribute significantly in the provision of specialist services. The bulk of anesthetic services in Malawi are provided by anesthetic clinical officers who have upgraded from being medical assistants.

### Allowances/per diems from meetings

Per diems and reimbursements for real and potential expenses incurred or expected to be incurred for attending workshops, seminars and conferences are important sources of extra-salary income for health professionals who have the opportunity to participate in such meetings. Some meetings provide 'reasonable' per diems which more than cover the expenses incurred, enabling health professional to have extra cash to take home. Even when the per diems are not as lucrative, savings can be made by, for instance, having a heavy breakfast or lunch when these are already included in the conference package and only having to pay for a lighter meal later.

Health professionals also make money from attendance to meetings through reimbursements for approved expenses. For example senior cadres may use their own or MoH vehicles and get fuel reimbursements. When using their own vehicles, over-estimation of fuel costs result in extra money to the health worker. When using an institutional vehicle and institutional fuel, the health professional may collect the reimbursement on behalf of the institution, but may not reimburse the institution. One reason given for such failure to take back the reimbursement is that there is currently no mechanism which would allow money to be deposited back into government accounts (for public service employees). The other reason given was that "*everyone knows about this*" and if you try to reinforce reimbursements to the institution, "*you are unlikely to get any support from anyone*."

The possibility of getting extra money from attendances at meetings is perceived to be the reason why some senior cadres are continually traveling from one meeting to another even when the agenda would have been more applicable to another health professional. This results in a perception among the junior cadres that they are only sent to the less lucrative meeting while their seniors are monopolizing attendance of out of station, better paying meetings.

Workers in senior management either at MoH or district level may also 'frog-leap' from one workshop to the other, even when the meetings are running concurrently. According to one health professional:

"What you have to do is just be available for sometime, register for the workshop and move on to the next workshop that you have been invited. In so doing, you are assured of the allowances."

It was also reported that when workshops are being conducted at the local institution, the senior management are all implicitly invited to attend to give their "blessing" to such meeting. One clinical officer indicated:

"The DHO is the overall in-charge of the district. Although s/he may not be physically at the workshop, he is spiritually with you and so s/he deserves the allowances."

The instances discussed above have potential to augment the health workers' salaries.

### Locum/part-time duties

Locum and part-time work for health professionals is one other way of making extra money. Ferrinho et al have described the practice of working in both the public and private health sector as "dual practice"(22) Professional qualifications and the particular needs of the clinic or hospital influence the cadre of staff employed for part-time duties. Large private hospitals may hire anesthetic clinical officers and radiographers but do not hire clinical officers or medical assistants in favour of doctors. Nurses are likely to be hired by smaller private clinics for part-time duties. Institutions normally serviced by doctors also hire locum doctors, some of whom are still doing their internships, contrary to Medical Council of Malawi (MCM) regulations. Although the MCM requires that a health professional should have clearance from their main employer for locum practice and an individual private practice license, many workers do not get the necessary employer's authorization neither do they have the MCM private practice license. The lack of awareness of such requirements and the high cost of the license underlie non-compliance.

Some health professionals, especially clinical officers, are proprietors of private clinics where they employ full-time or part-time staff, and also consult themselves out of hours. In some cases, this has attracted reprimands from the MoH Clinical services department who have felt that staff neglected MoH duties in favor of their personal interests at the private clinics. There is also a perception that some of the drugs and pharmaceutical supplies used at these private clinics owned by MoH employees have been pilfered from the public sector.

Some health professionals teach part-time at training institutions for para-medicals and nurses. An individual may even double their income this way as part-time pay may be lucrative.

### Change of work station

In order to make extra money some workers request transfer from one type of work environment to another either to facilitate off-duty clinic work or to allow travel to other places during off-duty days. Employment in a unit that only operates during day working hours allows for out of hours part-time work at a private facility. Employment in a district or central hospital in a ward where there are shifts, allows for several full days off in a month which can be used for personal income generation activities.

### Over-time work at public health facility

The shortage of health workers in most of government health facilities necessitates overtime work often paid for from user-fees. Mzuzu Central Hospital, for example makes provision to hire its own staff for part-time duties. Over-time creates opportunities for those who would otherwise have been off-duty, to work. Even though the money may not be substantial the income from overtime may comprise a significant proportion of a worker's income. There are however concerns as to the quality of health services that can be delivered by exhausted staff who are driven just by desire to make more money but may not be fit to work.

### Treating private patients during official work hours

Some health professionals who are fully employed in the public health sector treat private patients during work hours either at their private clinics or even within public health facilities. Patients are required to pay for services provided at the public health facility in exchange for being attended to much faster than would have normally been the case. Some health professionals do receive gifts from patients and/or relatives in order to facilitate procurement of services such as blood for transfusion, speedy consultations and clinical reviews.

### Double salaries and supplementation

Almost all health professionals are guaranteed employment into the public sector when they graduate from the training institutions in Malawi. In fact, students in the health professionals most often are identified for work stations before they receive their final examinations results. The public sector is therefore the natural first employer of potentially all health workers in the country.

From the public sector, health professionals then move to other job positions, including the training institutions, which can be described as quasi-state organizations as they are mostly run with state financial resources. Some health workers do not formally resign from the public sector and may continue to receive public sector salaries despite the fact that they are no longer employed.

A different situation occurs when a health professional is still employed within the public sector but has been transferred to another district. It may take a long time for that person to start receiving their salaries from the new unit. One administrator talking about workers who are still in the public sector but have moved to new work stations said:

"You do not normally remove a person from the payroll once they have transferred to another station because it takes long for them to start receiving their salaries from their new work station. Now, you do not know when they have started receiving at their new site. Many people continue receiving salaries from their previous work stations and I am discovering that some are also receiving salaries at their new station."

There are also reports of workers within health units and training institutions benefiting from a multiplicity of salary supplements from donors who are unaware that a worker is already in receipt of another supplement.

### Consultancy duties

Skilled health professionals are able to undertake consultancy research and/or training duties on behalf of the MoH, donor agencies, NGOs and other institutions. This is an important source of income especially for tutors and other faculty members within training institutions. Various compensation rates are in operation and a consultant can earn more for one day's work than all their monthly salary.

### Providing services to their places of work

Some health workers are suppliers of goods and services to their places of work. This privilege is mainly the domain of persons in management and administration. Such services include the provision of maintenance services and selling stationary and other consumables to the institution. Some health professionals either own firms or are associated with vendors who are suppliers to the organization. Some health professionals who may be associated with suppliers get commissions for goods and services provided to the organization. The existence of pre-qualification for suppliers does not seem to reduce this practice. One administrator said:

"It is mostly the accounts people that are into this. But the district health office administrator can stop this. But you create hatred if you do that."

### Involvement is small to medium sized businesses

Hospital environments are suitable sites for small-scale businesses such as selling soft drinks, doughnuts and other groceries. Some health professionals engage in these businesses as a means to earn extra income. Soft drinks might be stored in hospital food refrigerators or Expanded Program on Immunization (EPI) program or other hospital refrigerators and sold to patients, visitors and other staff. A nurse reported:

"My children sell soft drinks at home when they have knocked off from school. Since my house is close by, I can also keep an eye on what is going on there."

Health professionals also reported engaging in various other types of business not necessarily on health facility grounds. These involved running a minibus service and grocery shops.

### Minimizing expenditure

In this category will be discussed what health professionals do in order to reduce expenses for their livelihoods.

The general perception was that the cost of living was far beyond the means of their salaries. Strategies for minimizing personal costs include:

a) Walking to work for some part or the whole trip, rather than taking a bus

b) Sending children to public schools despite having negative attitudes towards these schools

c) Taking packed meals from home to eat at work or missing meals at work altogether

d) Using workplace resources for personal use e.g. use of work phones for personal calls and vehicles for personal errands.

### Choosing to work to rural areas

According to the Ministry of Health and Population Health Human Resources Plan, the majority of the health professionals in Malawi worked in urban areas [14]. Despite the rural-urban difference, which would suggest that the majority of health workers were in urban areas, some do choose to work in rural areas where the cost of living is perceived to be less than in urban areas. In rural areas, there usually are institutional houses which civil servants were given priority to rent. The rental charges are much lower than the market value of the houses. This privilege, although much more likely in the Ministry of Health, is also available to other government departments. The MoH is only second to the Ministry of Education in having its own accommodation which it rents to its employees.

### Perceptions of health professions towards survival practices

There were mixed feelings towards the various survival practices of health professionals in Malawi. These depended on the nature of the survival practice and whether individuals were assessing their own or another persons practice when attitudes tended to be more negative. There was a general feeling of futility that there was not much else the health professional could do to earn a livelihood, and that the health system itself was responsible for the creation of an environment in which corrupt practices were possible. However some health professionals expressed outright displeasure of practices that involved inflation of prices, earning commission on the purchase of hospital consumables, and the stealing and selling of medicines meant to be supplied for free to patients.

One expatriate administrator, commenting of reliance on allowances said:

"This is something I have observed here in Malawi and it is sad. But people get low salaries. Those that fund the health sector must also realize that if workers continue to be poorly paid, they (the workers) will find a way to supplement their salaries through various means. In the end it makes sense just to pool the resources for salaries and cheating will reduce."

## Discussion

Health professionals in Malawi are facing the challenges in their work in diverse ways ranging from being creative, unethical and in some cases legally questionable ways. One weakness of a qualitative study like this is the inability to document the extent of the problem, and in some cases, extremes may have preference to be reported.

The challenges faced by health professionals in Malawi deserve concerted action. This calls for both multi-sectoral and multi-pronged approaches involving the government, training institutions, health workers themselves, health worker associations/unions and the general public. Each these groups have a stake in ensuring that health professionals remain in the country to provide much needed health services.

The perceived discriminatory attitude against nurses stands out an area that may benefit from policy change. Currently, nurses can not become a district health officer (DHO) and are rarely appointed health center in-charge. The role of the DHO is mainly to provide leadership for all health programs in a district and is the chief administrator of these programs. The DHO is supported by persons trained in human resources management, accounts and administration. It is doubtful that a medical doctor is the only person who may posses the necessary skills and training for such roles. Even when this may have been cth case, that a clinical officer or medical assistant can be put to head or district or a health center when a degree nurse is available can not be justified. This is not in any way to demean the contribution of clinical officers and medical assistants, but rather to indicate that many nurses may be more qualified before the responsibilities are put on clinical officers and medical assistants.

The limited opportunities for supervision and overwhelming responsibilities shouldered by health professionals are crucial challenges in respect of human resources management. This could stem from the fact that human resources and in general, resource management does not feature significantly in the curricula of health worker training in Malawi. And yet, human resource managers may be these same health professionals with limited knowledge and skills about human resources. There are at least three options. Firstly, fully training human resources, non-health professional, could be hired to provide the necessary management of personnel. An alternative is to provide special training in human resources management to health professionals identified to manage staff. Finally, resources, including human resources management could be incorporated in the curricula of health professionals' training.

Malawi is implementing a decentralized health service. In part, this meant that regional health offices, each responsible for one of the three administrative regions of the country, were abolished. The thinking then was that the Ministry of Health headquarters would deal directly with districts. While this resulted in reduction of administrative bottlenecks, it also meant that supervision of the district was no longer done by the regions (which had been abolished), but rather the central office. It was later realized that there was just not enough human resources from the central office to carry out meaningful and regular supervision of the districts. As from 2004, the Ministry of Health created supervisory zonal offices that will be responsible for supervision of districts.

Supervision can not wait for zonal staff. Each level of service will need to have internal supervision and receive visits from the next hire level. The shortage of human resources and rationing of resources (transport, time) will continue to pose challenges to this need. It is of note however, that the National Tuberculosis Program has institute a formidable supervisory effort that is a model to other countries [23-25].

Many young doctors in Malawi aim to obtain post-graduate qualifications [26,27]. Until 2003, there were no local training programs and all post-graduate long-term specialist education for medical doctors was obtained abroad. Access was limited. Starting from 2003 however, it is possible to enroll in postgraduate course for medical doctors and nurses within Malawi. This has potential to encourage many deserving nurses and medical doctors to remain in Malawi. The downside of this progress is that some nurses and medical doctors will be better positioned to find opportunities abroad after obtaining higher qualifications. Participants reported that in some cases where a vacancy exists, recruitment methods are less transparent. This can be changed by requiring that all public sector jobs are advertised in the popular media and oversight by health professionals' associations is made. There may also be a complaints body that could have powers to investigate claims of wrong doing in recruitment of health professionals without victimizing the complainant.

Muula [28] has suggested that dual job-holding can, if properly administered, benefit both patients and health professionals. There is need to study how the current system is functioning in order to suggest how regulation can occur.

Some of the survival mechanisms such as stealing drugs and getting a double salary when one is doing one job and seeking payment for services that ought to be at no cost to patients are outright unethical. The relevant professional regulatory bodies need to strengthen their oversight role, and the law enforcement agencies ought to be involved in curbing these practices.

While many policy measures may be suggested for implementation, actual implementation is unlikely to occur at the same time. There is need to prioritize which policy measures need urgent attention, which ones may be implemented when more information is obtained and which one's may have limited impact.

The impact of HIV/AIDS on human resources is huge especially in heavily burdened countries such as Malawi. The World Health Report 2006 reports that death is the most common cause of attrition for health workers in Malawi [29]. While antiretrovirals are currently available for free in Malawi, there is need to increase accessibility. Just because the general public has access to HIV treatment does not mean that health professional who is also affected by being HIV infected themselves is accessing the service.

From April 2005, the Malawi government has instituted a salary supplementation to all health professionals working in the public sector. As a group, health professionals within the public sector are earning more than any other government employee on regular remuneration. It remains to be seen as to whether this initiative with slow down and reduce the out-migration of Malawian health worker to northern countries.

## Conclusion

The interest in the reasons behind health professionals' migration from developing to developed nation has sometimes led to researchers overlooking how those that have remained (in the developing nation) earn their living. We have reported that health professionals in Malawi, though facing significant challenges, are doing all they can to still make a living within a constrained environment. The current situation where health professionals in the public/civil service earn more than other workers probably will go a long way in sending the message that health professionals are valued in Malawi.

## Competing interests

The author(s) declare that they have no competing interests.

## Authors' contributions

ASM conceived the study, designed the survey instruments, supervised data collection and contributed to data analysis, drafted manuscript and approved final copy of manuscript. FCM contributed to design of survey instruments, collected data, contributed to data analysis and critical review of manuscripts draft and approved the final draft.

## Appendix 1: Interview guide for health workers

The research assistant (RA) is required to introduce themselves and the purpose of the visit. After this, the RA should obtain verbal consent. If the health worker chooses not to participate, kindly thank the person and move on to the next health worker that is lined up for interviews.

1. What challenges do health workers face in this country?

2. How could (name each of the challenges suggested) be solved?

3. Could you describe for me the various sources of income that you have?

4. Is there anything else that you do from which you obtain money or goods?

5. What else apart from those that you have mentioned?

6. How do some of the health workers that you have known obtain income?

7. Let us start with your sources of income, how do you perceive such methods? Do you like them or you have some reservations?

8. Probe more on the perceptions of the methods by the health workers?

9. How do you think other people perceive such kind of income or resource generation?

10. The issue of loss of health staff has attracted public attention. Why do you think health workers eventually leave the country?

11. What do you think must be done in order to make health workers remain in the country? What must be done to enable health workers to remain at their place of work?

12. What is already being done in order to retain health workers?

13. What must the government do to enable retention of health workers?

14. What should the health workers themselves do to ensure that health workers remain in the country?

## Appendix 2: Interview guide for administrators and policy makers

The research assistant (RA) is required to introduce themselves and the purpose of the visit. After this, the RA should obtain verbal consent. If the administrator/policy maker chooses not to participate, kindly thank the person and move on to the next appointment that is lined up.

1. Could you tell me the challenges that health workers are facing in Malawi

2. What other challenges do the health workers meet?

3. If the participate has not mentioned some of the following (shortage of health workers, shortage of hospital supplies, lack of promotion or clear career path, increased work load, HIV and AIDS, infection risk, low remuneration), please suggest and obtain a response whether these are perceived by the respondent as notable challenges.

4. How could these challenges (name each of the suggested challenges) be dealt with?

5. What is the government dealing with these challenges?

6. What is your perception of the brain drain phenomenon?

7. Why are health workers leaving Malawi to work elsewhere?

8. What is being done to reduce the brain drain?

9. What must be done to stem the drain of health personnel?

10. What is your perception of these measures? If question seems not clear to respondent, ask what is his or her evaluation. Will the measures work, not work, easy to implement or difficult to implement? What may pose as challenges in the implementation of the measures.

11. Despite the fact that health workers receive poor remuneration, how are they then surviving?

12. What is you perception on (name a way of earning income e.g. over-time)

13. What policy frameworks are available to address the human resources shortages (and brain drain) in Malawi?

14. What is your assessment of this (name the policy)? Will it be able to deliver the desired goal? What challenges do you think presently exist? What challenges do you think the future will present?

## Pre-publication history

The pre-publication history for this paper can be accessed here:


